# Tunnel Encapsulation Technology for Durability Improvement in Stretchable Electronics Fabrication

**DOI:** 10.3390/mi9100519

**Published:** 2018-10-14

**Authors:** Kangmin Leng, Chuanfei Guo, Kang Wu, Zhigang Wu

**Affiliations:** 1 State Key Laboratory of Digital Manufacturing Equipment and Technology, Huazhong University of Science and Technology, Wuhan 430074, China; lengkangmin@hust.edu.cn; 2 Department of Materials Science and Engineering, Southern University of Science and Technology, Shenzhen 518055, China; guocf@sustc.edu.cn

**Keywords:** stretchable electronics, tunnel encapsulation, Polyvinyl Alcohol, durability

## Abstract

Great diversity of process technologies and materials have been developed around stretchable electronics. A subset of them, which are made up of zigzag metal foil and soft silicon polymers, show advantages of being easy to manufacture and low cost. However, most of the circuits lack durability due to stress concentration of interconnects entirely embedded in elastic polymer silicone such as polydimethylsiloxane (PDMS). In our demonstration, tunnel encapsulation technology was introduced to relieve stress of these conductors when they were stretched to deform in and out of plane. It was realized by dissolving the medium of Polyvinyl Alcohol (PVA), previous cured together with circuits in polymer, to form the micro-tunnel which not only guarantee the stretchability of interconnect, but also help to improve the durability. With the protection of tunnel, the serpentine could stably maintain the designed shape and electrical performance after 50% strain cycling over 20,000 times. Finally, different materials for encapsulation were employed to provide promising options for applications in portable biomedical devices which demand duplicate distortion.

## 1. Introduction

With a significant development of fabrication processes and patterning technologies, stretchable electronic devices, based on inorganic materials, have achieved high performance in bioinspired and biointegrated systems in the past few years [1,2,3]. To achieve conformal contact with human skin and maintain stable electrical performance, these devices need to endure strain and stress from many repetitive deformation movements in or out of plane. Special designed structures such as zigzag and serpentine interconnects [4,5,6] provide the allowance for deformation. This brings the fabrication feasibility for stretchable electronics based on thin inorganic materials [7,8,9]. Moreover, the prestrain strategy [10] has been reported extensively. It significantly increased the stretchability of interconnects of different shapes for biomedical and healthcare applications has been reported in recent years [11]. Among those studies, very thin metal wires were patterned on the soft substrates through evaporation or transfer printing [12,13]. The finished wires and substrates were encapsulated by pouring a thin soft polymer directly to form a protection layer [5,14,15]. During these processes, encapsulation technologies are closely related to the lifetime and durability when these devices are up against mechanical damage in practical application. The differences of modulus and morphology between metal wires and polymer matrix bring problems when deformation happens. For example, the out-of-plane wavy and 3D buckling deformation for metal wires are constrained by those closely embedded polymers as there are no spaces for these movements [6,14]. They come out of the reducing of desirable stretchability and durability of the systems. Recently, an option involving microfluidic suspensions in thin elastomeric enclosures at system level was proposed [16]. However, this technology was faced with fluid leakage and shape distortion under large deformation. However, research focusing on the stretchability and durability of interconnects with encapsulation needs further supplementary research and perfection [17,18,19].

Here, we present a new encapsulation technology for the life time improvement of stretchable interconnects. A three-dimensional tunnel space was made to enable slipping and buckling for deformed interconnects. We describe the newly invented encapsulation technology of embedding the serpentine interconnect in three-dimensional tunnel, called tunnel encapsulation. In this study, the micro-tunnel space was realized by dissolving PVA, previous cured together with interconnects in polymer. Nevertheless, patterning the conductive metal films on PVA is a challenge. Previous studies reported some techniques including lithography and ion etching thin films onto PVA [20,21,22]. These technologies provide patterned films with high accuracy but there are problems such as complicated processes and high cost associated with cleanroom facilities. In addition, greater demands were being placed on the processing equipment, the operator and operating environment. In our technology, metal film and PVA film were directly patterned by laser processing and then bonding with each other for the stickiness of wetting PVA. Experimental studies and finite element analyses (FEA) revealed the important features of our technology and their dependence on key design parameters to predict the durability of the electronic components for a periodic deformation. Different interconnect patterns and polymer matrix materials were applied to evaluate the universality of this new encapsulation and it was proved to be valid. Remarkably, even when stretched with 50% strain, the electrode was still able to recover its original shape and maintain good electrical conductivity. An appropriate dimension and lubricating fluid for the tunnel which enclose the interconnects are critical. In particular, fixing-pillar was proposed to strengthen local stiffness in the arc regions of the serpentine, which could strongly enhance the lifetime of the interconnects. Overall, we provided experimental and simulation results that can fabricate a high-performance electronic device in encapsulation with excellent stretchability and durability. The fundamental difference between the direct encapsulation and tunnel encapsulation is the introduction of the three-dimensional tunnel. For direct encapsulation, interconnect embedded in polymer will encounter severe stress concentration when stretched. The severe stress concentration results in the limited stretchability and lifetime of the interconnect. For tunnel encapsulation, stress concentration is alleviated when stretched at the same degree of elongation for the introduction of tunnel space. Meanwhile, the interconnect would slide and buckle in the tunnel to release stress. With resizing the tunnel, the maximum stretchability and fatigue performance of interconnect can be modified. Therefore, the tunnel encapsulation helps to improve the maximum stretchability and lifetime of the system.

## 2. Materials and Technologies

### 2.1. Preparation of PVA Membrane

A 9:1 mixture of deionized water and granular Polyvinyl Alcohol (PVA) (87.0∼89.0% hydrolyzed, Mw 130,000 g/mol, Shanghai Macklin Biochemical Co., Ltd., Shanghai, China) was heated in a glass beaker at 75 ${}^{\circ }$C for 1 h with stirring, Then, the mixture was kept at room temperature (25 ${}^{\circ }$C) with vigorous stirring for about 30 min to obtain 10 wt.% PVA aqueous solution. We fabricated pristine PVA membranes by casting the 10 wt.% PVA aqueous solution onto a culture dish and drying the solutions at 75 ${}^{\circ }$C for 2 h.

### 2.2. Preparation of PDMS

Poly-dimethyl siloxane (PDMS) (Sylgard 186, Dow Corning (Midland, MI, USA)) was chosen as the elastomeric substrate to carry the patterned metal on top. The two-part liquid components (PDMS base and curing agent) were mixed with weight ratio of 10:1 in a plastic cup successively and mixed together manually for 5 min with a glass rod. Prior to putting into use, vacuumization (10 min) and refrigeration (about −18 ${}^{\circ }$C for 1 h) were used to eliminate the air bubbles.

### 2.3. Fabrication of Stretchable Electronics in Tunnel Encapsulation

**Figure 1:**
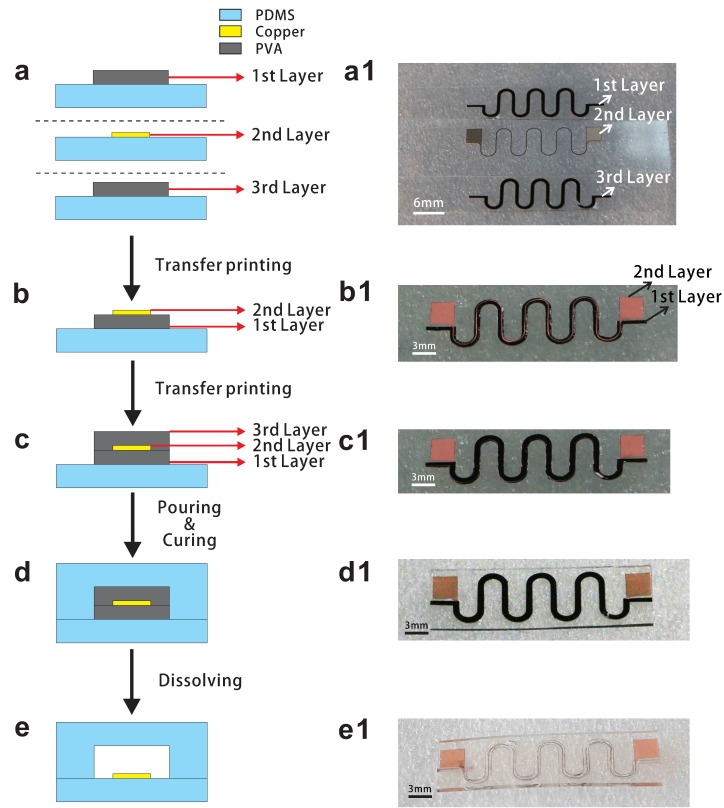
Schematic diagram of the tunnel encapsulation fabrication process. (**a**) Patterned copper foil and PVA membrane, where the first layer and the third layer have the same dimension. (**a1**) Optical image of patterned copper film and PVA. (**b**) Metal circuit was transferred to the patterned PVA. (**b1**) Optical image of the double layer on the receiver substrate. (**c**) Another patterned PVA was transferred to the top of the double layer structure. (**c1**) Optical image of the sandwich structure. (**d**) PDMS was poured to direct encapsulate the sandwich structure and cured at 75 ${}^{\circ }$C for 2 h. (**d1**) Optical image of the sandwich structure encapsulated with PDMS. (**e**) Both ends of the laminate strip were cut to expose the entrance and exit for dissolving the PVA around the Copper circuit. (**e1**) Optical image of the sample after dissolving the PVA.

A schematic of the tunnel encapsulation process is shown in Figure 1. Firstly, PDMS was poured on top of a ceramic carrier at 90 ${}^{\circ }$C for 10 min to form the donor substrate. In our demonstration, a commercial electrolytic copper foil (EQ-bccf-9u, thinness: 9 μm, Shenzhen Kejing Star Tech, Shenzhen, China) was adhered to the donor substrate. Similarly, two solidified PVA membranes (the width was 1000 μm and the thickness was 50 μm; we dyed the PVA solution with black ink for visibility) were adhered to another two donor substrates. Afterwards, a UV laser marker (HGL-LSU3/5EI, Wuhan Huagong Laser Engineering Co., Ltd., Wuhan, China) was used to pattern the copper Foil and PVA membrane, respectively [23]. The required pattern and unnecessary parts were cut off by UV laser. The patterns of copper and PVA are shown in Figure S1. After tearing off the obsolete PVA membrane and copper foil (Figure 1a), a patterned PVA film was transferred from a donor to a receiver substrate (made at 90 ${}^{\circ }$C for 4 min) for the different stickiness of substrate. Then, the required copper circuit was wetted with deionized water (place the copper over a bottle filled with 100 ${}^{\circ }$C deionized water about 15 s) and put into contact with the copper and PVA without external pressure for 5 s. Afterwards, the copper circuit was sticking to the patterned PVA due to stickiness increasing of wetting PVA (Figure 1b). In other words, the copper was transferred from the donor to the patterned PVA which was on the receiver substrate. Same as above procedure, another donor substrate covered with patterned PVA was wetted with deionized water, and the patterned PVA was transferred from the donor to the top of the double layer. After stacking up, the water would dissolve the interface of patterned PVA. The copper was embedded in PVA when constructing the PVA/copper/PVA sandwich structure (Figure 1c). Then, the PDMS was poured to directly encapsulate the sandwich structure and cured for 2 h at 75 ${}^{\circ }$C (Figure 1d). The PDMS/sandwich structure/PDMS laminate was cut into strips, each strip has one patterned meander metal interconnect encapsulated in the stretchable substrate. The edges of strips were cut to expose the entrance and exit. At the final step, these strips were placed in deionized water for 24 h at 75 ${}^{\circ }$C to dissolve the PVA which enclosed those copper circuits (Figure 1e). The residual PVA was washed away by an injection needle filled with deionized water. From a practical point of view, the entrance and exit of these strips should be sealed to prevent the corrosion of the circuits. The critical point of the fabrication is the intimate bonding of PVA and copper, and it can be assured by vacuuming when pouring the upper PDMS before curing. Nevertheless, it may affect the shaping of tunnel.

### 2.4. Stretching and Electrical Test

All the strips were clamped by two pieces of thin PDMS on both sides, and a high-frequency fatigue testing machine (E1000, Instron, Boston, MA, USA) stretched the sample to a specified displacement specified through program for cyclic stretching at 2 Hz. The ends of the interconnects were welded to connect the test electrode; a digital multimeter (34,461A, KeySight Technologies, Santa Rosa, CA, USA) was used for electrical test.

### 2.5. Finite Element Analysis

ABAQUS commercial software (ABAQUS6.14, ABAQUS Inc., Palo Alto, CA, USA) was used to study the mechanics response of tunnel encapsulation and direct encapsulation. PDMS was modeled by the hexahedron element (C3D8R), while the interconnect was modeled by the shell element (S4R). 22,749 elements for silicone and 1419 elements for interconnects were used to conduct FEA modeling after grid independence testing. Displacement boundary conditions were applied to both edges of the system to apply different levels of stretching and refined meshes were adopted to ensure the accuracy. ABAQUS/Explicit was applied to analyze the deformation and normal stress distribution of interconnects with tunnel encapsulation and direct encapsulation.

## 3. Results and Discussion

### 3.1. The Performance during Stretching

**Figure 2:**
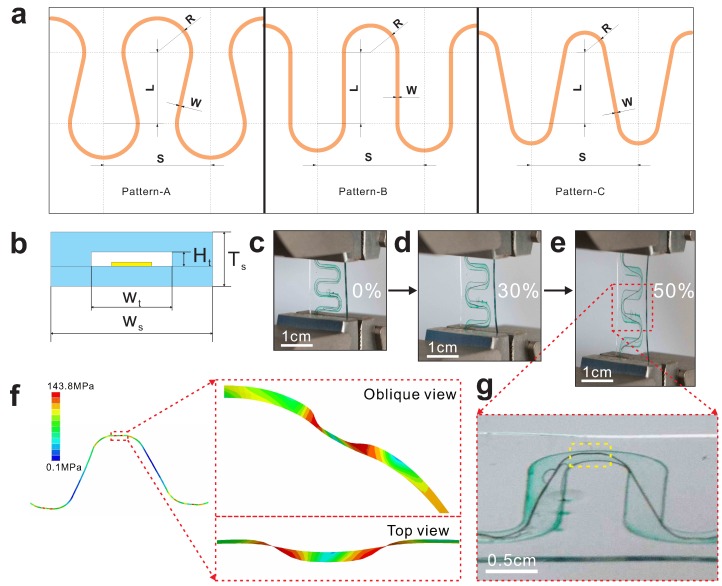
(**a**) Three different patterns of serpentine interconnects. (**b**) Cross-sectional illustration of representative layers in a hollow structure with embedded stretchable interconnects which collapse in the tunnel. (**c**–**e**) Optical images of the sample before stretched, morphology at 30% and 50% applied strain, respectively. (**f**) FEA results of morphology at 50% strain. (**g**) Magnification of slipping and buckling region.

Figure 2a shows three different patterns of serpentine interconnects which consist of two periodic unit cells of two straight lines and two arc of circles, and the straight lines are tangent to the arc. R is the outer radius, S is the space of two unit cells, L is the center distance of two arc along the longitudinal axis, and W is the width of the copper trace. The center distance of these patterns was fixed to set a limit for the dimensions of these circuits. Therefore, R is the critical parameter of these pattern, which controls the shape of the pattern. We obtained horseshoe design (Pattern-A, R = 1.95 mm, S = 6 mm, L = 4 mm, W = 100 μm), semicircle design (Pattern-B, R = 1.55 mm, S = 6 mm, L = 4 mm, W = 100 μm) and the snakelike design (Pattern-C, R = 1.15 mm, S = 6 mm, L = 4 mm, W = 100 μm). Let Ht denote the height of the tunnel made of PVA; Wt the width of the tunnel; Ws the width of the strip; and Ts the thickness of the strip. Through the thickness, the strip has a layered structure (Figure 2b) from bottom (substrate/serpentine/encapsulation layer) to top. A sample (Ht = 100 μm, Wt = 1000 μm, Ws = 1 cm, Ts = 500 μm) with serpentine designed Pattern-B is presented sequentially to illustrate the characteristic of tunnel encapsulation. Figure 2c shows the original shape of the interconnect with tunnel encapsulation before stretched and the tunnel was filled with green fluid to identify the deformation of tunnel. Figure 2d exhibits the serpentine interconnect at 30% applied strain obtained from dynamic mechanical analysis. Figure 2e exhibits the serpentine interconnect at 50% applied strain. Figure 2g illustrates the interconnects slip and buckle in the tunnel when stretched. It is shown that buckling occurred in the arc region highlight with the yellow frame and it is identical with the FEA result (magnification in Figure 2f). The corresponding FEA predictions on buckling deformation of serpentine interconnects illustrate that the tunnel provides enormous space for the interconnects to relieve stress with slipping and buckling.

**Figure 3:**
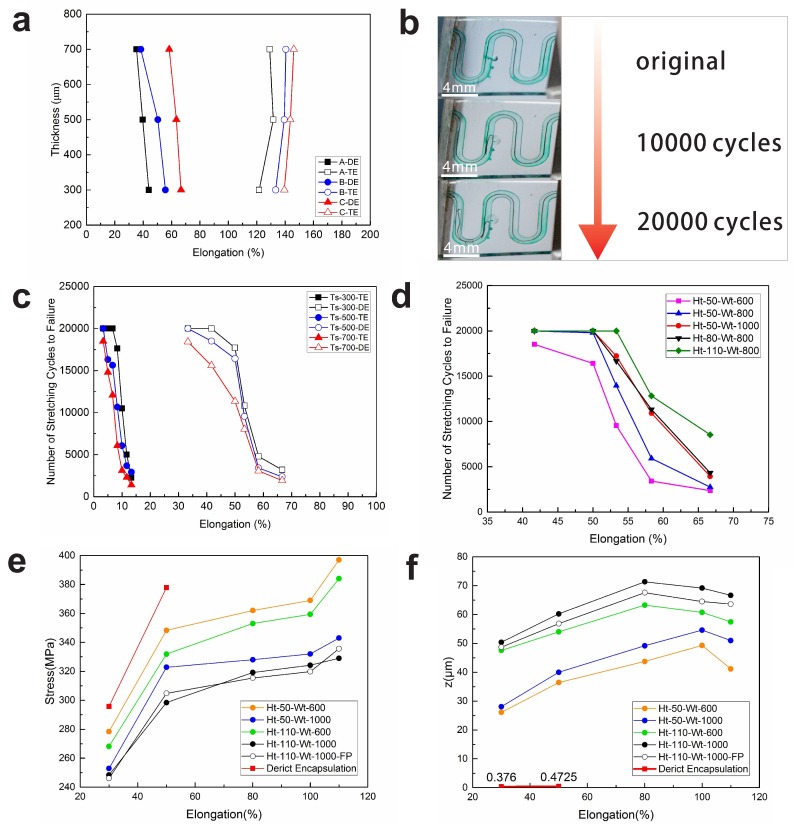
(**a**) Maximum stretchability when the serpentine (different patterns) encapsulated in different technologies with different sample thicknesses Ts from 300 to 700 μm and the width of these samples is 600 μm. (**b**) Reversibility illustration of serpentine in tunnel encapsulation (Ht = 100 μm, Wt = 1000 μm, Ws = 1 cm, Ts = 500 μm). (**c**) Number of stretching cycles to failure when the serpentine encapsulated in different technologies with different sample thicknesses Ts from 300 to 700 μm and the width of these samples is 600 μm. (**d**) Experimental results for different width and height of tunnel in system (Ts = 500 μm). (**e**,**f**) FEA predictions on stress and relative displacement in z direction of serpentine interconnects under stretching.

We performed elongation test with both encapsulation technologies to investigate the effect of encapsulation technology on maximum stretchability. Three serpentines of different patterns were encapsulated in PDMS with both technologies to confirm the effect of patterns on maximum stretchability. In addition, to evaluate the effect of thickness of encapsulation layer, we made samples with three different thicknesses (300 μm, 500 μm, and 700 μm). The results of the experiment are shown in Figure 3a, where the direct encapsulation and tunnel encapsulation are abbreviated to DE and TE, respectively. For direct encapsulation without further processing, the maximum elongation is limited by localized fracture of the copper embedded in silicone, and the interconnect encounters severe stress concentration originating from constraints of surrounding PDMS (Figures S2 and S3). The FEA result in Figure 3e illustrates the stress of interconnect with direct encapsulation increase dramatically (stress at 80–110% strain exceed the maximum stress, hence values are not shown in the graph). For tunnel encapsulation, the serpentine interconnect was freestanding in the tunnel except the two ends. Upon stretching of the whole system, the interconnect is dragged from the two ends, geometrical opening in arc segment increases to accommodate the elongation, the straight part and the arc segment undergoes both stretching and bending. As the applied strain exceeds a critical value, lateral buckling occurs to reduce the strain energy, which helps to alleviate stress concentration of interconnects. Figure 3e shows that the stress of interconnects with tunnel encapsulation is considerably lower than interconnect with direct encapsulation. Besides, the platform period of stress with tunnel encapsulation in Figure 3e proved that the outer stretching is accommodated by the sliding and buckling behavior of interconnect in tunnel (Figures S4–S6). It is shown that Pattern-C owns the best stretchability in both encapsulation technologies. A larger curvature in the arc region become closer to a straight line. That means Pattern-C serpentine has a larger redundancy to deform with both technologies as the curvature of Pattern-C is the smallest. The stretchability of the system decreases as the thickness of the strip increases when encapsulated directly (Figure 3a) since the interconnects suffer severer mechanical constraints at elongation. On the contrary, the trend of the curve of tunnel encapsulation is opposite to the direct encapsulation. In tunnel encapsulation, PDMS around the serpentine broke first before the copper rupture as the tunnel would decrease the strength of the strip. As a result, with the same dimension of tunnel, the degradation of a thinner strip will become worse.

Figure 3b illustrates the reversibility of serpentine (Pattern-B, Ht = 100 μm, Wt = 1000 μm, Ws = 1 cm, Ts = 500 μm) in tunnel encapsulation. The serpentine can recover its original shape roughly under 50% strain after 20,000 cycles at 2 Hz. As the interconnects hang in the air deformed, typically by out-of-plane buckling and in-plane bending, they will lose the original shape when the stress is relieved. With tunnel encapsulation, the surrounding silicone matrix will mechanically constrain deformation of the interconnects in the case of the serpentine coming loose in the tunnel space. In the experiment, as long as the applied strain was under maximum strain for all kinds of patterns and thicknesses, the system could ensure reversibility in its mechanics.

### 3.2. The Effect of Stress Concentration on Durability

We studied the influence of our technology on the durability with the same pattern design (Pattern-B). We performed cyclic loading experiments with a focus on the lifetime of the serpentine, defined as the number of stretching cycles to failure in the interconnect. Figure 3c shows the number of stretching cycles to failure in the interconnect versus applied strain for different encapsulation technologies and different system thicknesses (300 μm, 500 μm, and 700 μm). For the direct encapsulation, the differences on lifetime with corresponding elongation are caused by the fact that the encapsulation would further constrain the interconnects from deforming out of plane if elongation is increased. Meanwhile, stress concentration occurs in the arc region of the serpentine and the phenomenon becomes rather obvious with increasing elongation. The FEA result (Figure 3e,f) also verifies the hypothesis. Remarkably, the lifetime of serpentine improved tremendously by the tunnel encapsulation. Likewise, the differences on lifetime with corresponding elongation are caused by further constraints and severer friction contact. Note that the outstanding improvement on lifetime with tunnel encapsulation stem from the introduction of tunnel space. It offers the activity space for the interconnects to slide and buckle in the space to relieve stress at elongation. In addition, the tunnel isolates the interconnect from surrounding PDMS and helps to decrease the friction of interconnect when stretching. The result in Figure 3c reveals that a thinner system would improve the lifetime of the serpentine as the constraints of a thinner encapsulation layer are weaker. It can be concluded from those results that the lifetime of the serpentine decreases significantly with increasing system elongation. Besides, both the tunnel encapsulation technology and a thinner encapsulation layer help to improve the lifetime of the interconnects.

The available space depends on the width and the height of the tunnel. Thus, a comparison of different width tunnel encapsulation is presented in Figure 3d. It is obvious that the lifetime increase with a wider tunnel. It might be attributed to a broader space enabling a greater degree of slipping and buckling of the interconnects (see green and black line in Figure 3f). In addition, the result depicts the relationship between lifetime of interconnects and the height of tunnel. The lifetime increased as the height increased, which gives rise to a larger space to buckle for the serpentine in the tunnel under stretching, which is a desirable characteristic for cycling stretch. The FEA result (see orange and green line, blue and black line in Figure 3f) shows that the greater heights enable the interconnects to have a larger displacement in z direction, which means the stress relieved when buckling in the direction (see orange and green line, blue and black line in Figure 3e). The displacement in z direction would decrease after a specified value of strain for the severer buckling (Supplementary Materials, Figure S7). Therefore, the durability of the interconnects can be improved by increasing the width and height of the tunnel.

**Figure 4:**
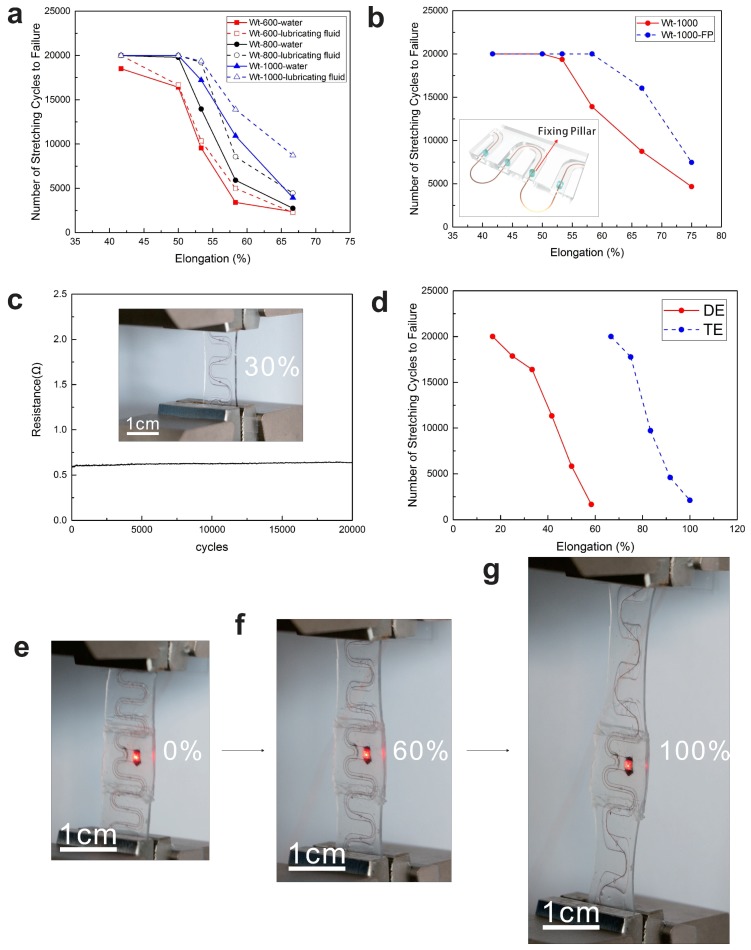
(**a**) Experimental results for effect of lubricating fluid on lifetime of samples with different tunnel widths (sample thickness Ts = 500 μm and height of the tunnel Ht = 100 μm). (**b**) The corresponding improvement in lifetime when adopt fixing-pillars (300 μm in diameter) in the tunnel filled with lubrication fluid (Ht = 100 μm, Wt = 1000 μm, Ws = 1 cm, Ts = 500 μm) and schematic illustration of fixing-pillars (insets). (**c**) Experimental results of resistance change with fixing-pillars in tunnel (Ht = 100 μm, Wt = 1000 μm, Ws = 1 cm, Ts = 500 μm) and optical images of deformation with pillars in tunnel at 30% strain. (**d**) Experimental results of serpentine with direct encapsulation and tunnel encapsulation in Ecoflex. (**e**–**g**) Optical images of device before stretched and morphology at 60% and 100% applied strain, respectively.

The tunnel was filled with a silicone oligomer (Sylgard 184, without curing agent) using an injection needle to lubricate the contact surface and reduce the friction between interconnects and silicon. The effect of injected fluid was quantitatively studied by experimental tests, as shown in Figure 4a. It is clear that the lifetime of the interconnects was improved as the lubricating fluid reduced the nonspecific adhesion between the interconnects and the tunnel. Besides, it is hydrophobic, expelling moisture from the package, and is optically transparent. It is remarkable that all the experiments were done at once after dissolving. It can be seen that the lifetime of interconnect in water-filled tunnel declines rapidly and the water may corrode the copper interconnect. On the contrary, interconnect in silicone oligomer-filled tunnel sustains excellent fatigue performance without the risk of corrosion. The contact between the interconnects and the wall of the tunnel will definitely influence the lifetime of the serpentine. Apparently, the height of the tunnel plays an important role in optimization as described above. Taking the interconnect itself into account, diminishing the out-of-plane displacement when stretched may contribute much to the lifetime of the system. Here, we made several flexible fixing-pillars in the tunnel to fix the position of the interconnect. The results are shown in Figure 4b. It is important to note that the experiment is based on the lubricating fluid. It was observed that the lifetime increased enormously with introduction of fixing-pillars. We reduced the local stiffness of the arc region of the serpentine so that the deformation in the arc part would be alleviated, and the probability of contacting between interconnects and the wall of the tunnel diminished accordingly. Of course, the pillars are located in the minimum deformation part of the interconnects. For the patterns we used, it was the middle part of the straight segments of the serpentine structure. The FEA result shows that the fixing-pillar help to decrease displacement in z direction while maintaining similar degree of stress (Figure 3e,f). Besides, it just needs to modify the pattern of the PVA a little bit to be an effective technology that is able to improve the durability of interconnects when applying this new technology of encapsulation.

### 3.3. Electrical Performance

The stretchable serpentine shows excellent cycling stability in Figure 4c. The parameters of the sample are Ts = 500 μm, Wt = 100 μm, and Ht = 100 μm, after 20,000 cycles at 50% applied strain, the interconnects still exhibited an almost constant value. Note that no visible cracks formed in the interconnects when inspected under an optical microscope, and the system can maintain stable electrical performance under repetitively large deformation.

### 3.4. The Universality of Tunnel Encapsulation Technology

To study the universal property of this new encapsulation, Ecoflex was selected as encapsulation material to test the durability of the system in Figure 4d. The lifetime of the serpentine encapsulated with Ecoflex improved enormously compared with PDMS, and it is suitable for tunnel encapsulation. In addition, it was observed that the interconnects reached its rupture strain before the Ecoflex broke. The stretchability is up to 200% for the serpentine structure of the metal, but not the ultimate limit for interconnects which are not encapsulated. The interconnects in the tunnel can recover the original shape practically even at the maximum elongation for the constraints of the tunnel.

## 4. Device Display

A long serpentine interconnect encapsulated in Ecoflex with tunnel encapsulation is presented in Figure 4e–g. An LED was integrated in the device, and fixing-pillars were applied in the tunnel. The parameters of the device are Ts = 500 μm, Wt = 1000 μm, and Ht = 150 μm, and the interconnect with tunnel encapsulation can sustain electrical performance when stretched to reach 100% strain. In this device, a piece of PDMS was attached over the central part to protect the connecting of the LED chip and interconnects. When the system was elongated, the interconnects of both end endure the whole elongation while the middle part would sustain the original shape. Considering integration of microchip, it is worth exploring placing a microchip in the three-dimensional tunnel to further alleviate stress concentration on the chip.

## 5. Conclusions

In summary, we report a technology of tunnel encapsulation to improve the lifetime of stretchable interconnects A tunnel is formed from dissolution of PVA in deionized water which can help alleviate the stress concentration by provide the space for the sliding and buckling of interconnects. Our tunnel encapsulation confers superior properties compared to direct encapsulation, namely: (1) exceptional stretchability and durability, our approach offers a three-dimensional activity space for the interconnects to buckle, twist and stretch, the stretchability can be up to 200% when the interconnects encapsulated in Ecoflex; (2) excellent stability of electronics conductivity, the interconnects can sustain excellent electrical performance after 20,000 cycles at 50% applied strain; and (3) ease of patterning, the pattern of the tunnel can be readily changed by modifying the pattern of PVA. This new encapsulation has a good application prospect for consumer wearable electronics.

## Supplementary Materials

The following are available online at http://www.mdpi.com/2072-666X/9/10/519/s1, Figure S1: (a) Pattern of copper; (b) Pattern of Polyvinyl Alcohol (PVA), Figure S2: Stress distributions and strain contours of the interconnects with direct encapsulation at 30% elongation, Figure S3: Stress distributions and strain contours of the interconnects with direct encapsulation at 50% elongation, Figure S4: Stress distributions and strain contours of the interconnects with tunnel encapsulation at 30% elongation, Figure S5: Stress distributions and strain contours of the interconnects with tunnel encapsulation at 50% elongation, Figure S6: Stress distributions and strain contours of the interconnects with tunnel encapsulation at 100% elongation, Figure S7: Stress distributions and strain contours of the interconnects with tunnel encapsulation at 110% elongation.
