# Supplementary material for: Tunnel Encapsulation Technology for Durability Improvement in Stretchable Electronics Fabrication
Source: Micromachines (Basel). 2018 Oct 14;9(10):519. doi: 10.3390/mi9100519 (PMC6215241; doi:10.3390/mi9100519)
Supplement: Supplementary file 1 [file micromachines-09-00519-s001.pdf]

Supplementary Materials: Tunnel Encapsulation Technology for Durability Improvement in Stretchable Electronics Fabrication

Kangmin Leng, Chuanfei Guo, Kang Wu and Zhigang Wu

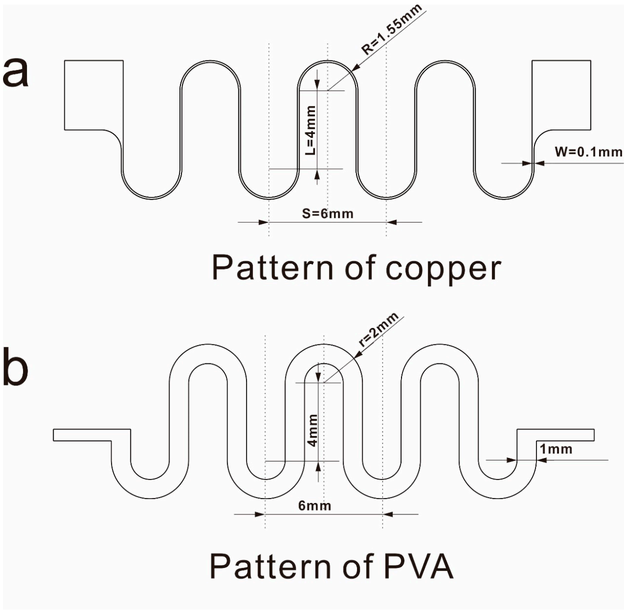

Figure S1. (**a**) Pattern of copper; (**b**) Pattern of Polyvinyl Alcohol (PVA).

Finite element analyses (FEA):

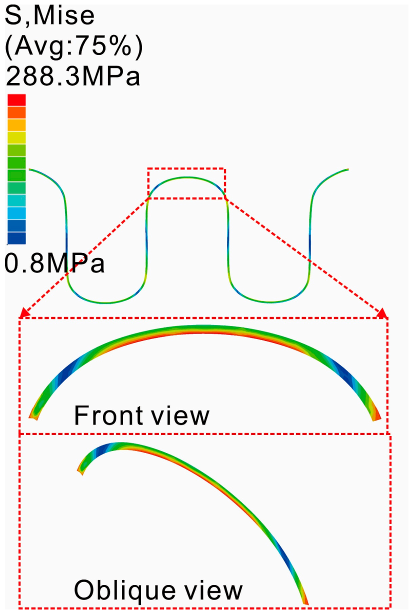

Figure S2. Stress distributions and strain contours of the interconnects with direct encapsulation at 30% elongation.

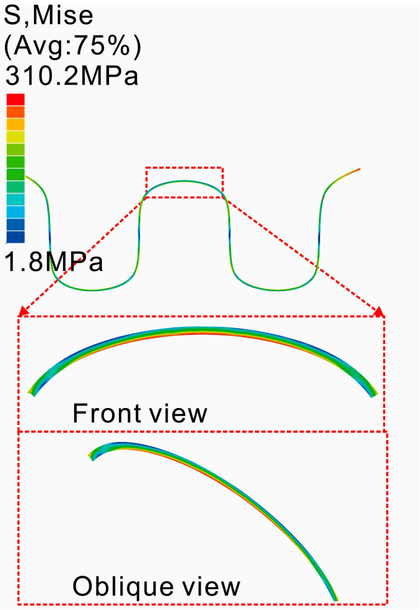

Figure S3. Stress distributions and strain contours of the interconnects with direct encapsulation at 50% elongation.

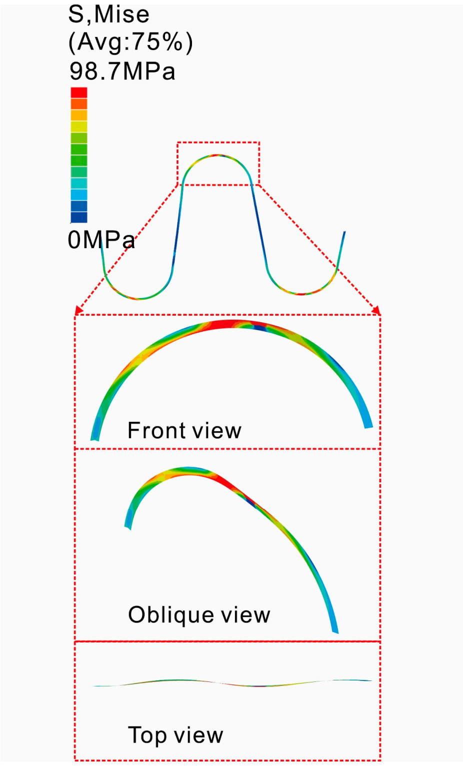

Figure S4. Stress distributions and strain contours of the interconnects with tunnel encapsulation at 30% elongation.

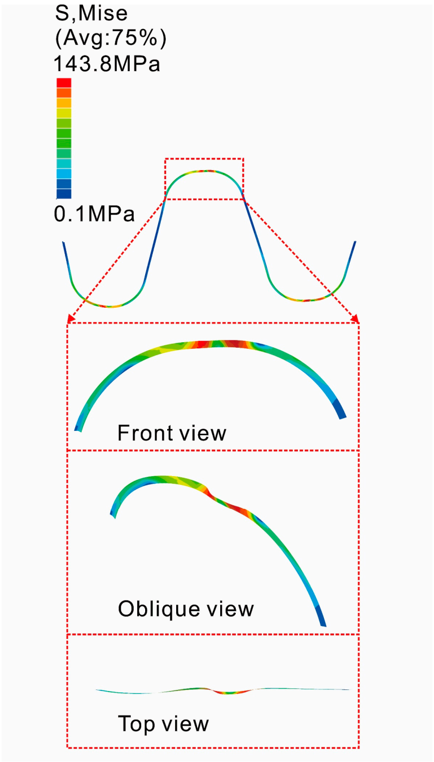

Figure S5. Stress distributions and strain contours of the interconnects with tunnel encapsulation at 50% elongation.

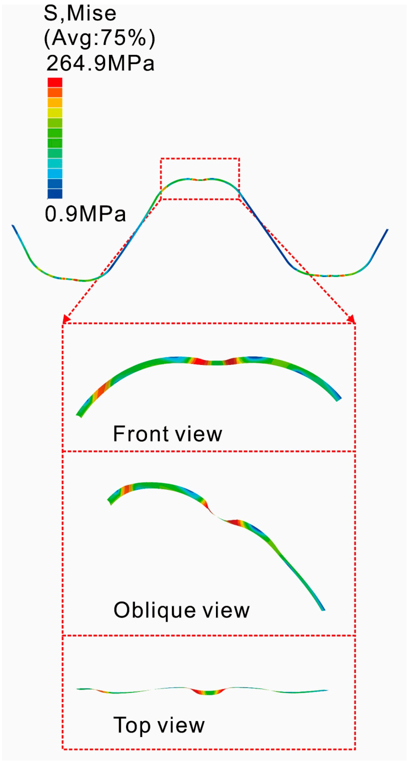

Figure S6. Stress distributions and strain contours of the interconnects with tunnel encapsulation at 100% elongation.

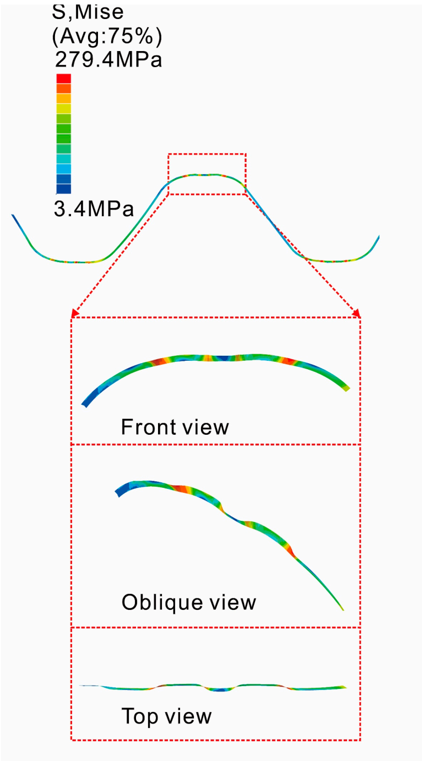

Figure S7. Stress distributions and strain contours of the interconnects with tunnel encapsulation at 110% elongation.
